# Supplementary material for: ExpressAnalyst: A unified platform for RNA-sequencing analysis in non-model species
Source: Nat Commun. 2023 May 24;14:2995. doi: 10.1038/s41467-023-38785-y (PMC10209063; doi:10.1038/s41467-023-38785-y)
Supplement: Supplementary file 1 — Supplementary Information [file 41467_2023_38785_MOESM1_ESM.pdf]

Supplementary Information for:

ExpressAnalyst: a unified platform for RNA-sequencing analysis in non-model species

Peng Liu[1]*, Jessica Ewald[1]*, Zhiqiang Pang[1], Elena Legrand[1], Yeon Seon Jeon[1], Jonathan Sangiovanni[1], Orcun Hacariz[1], Guangyan Zhou[1], Jessica A. Head[1], Niladri Basu[1], Jianguo Xia[1]

[1] Faculty of Agricultural and Environmental Sciences, McGill University, Ste-Anne-de-Bellevue, Canada

* These authors contributed equally to the work.

Correspondence:
Email: jeff.xia@mcgill.ca (J.X.)
Tel.: +1-(514) 398-8668 (J.X.)

Supplementary Note 1

"Raw Data Processing" Module in ExpressAnalyst

Raw data processing can be performed online or locally, using a Docker implementation. If users select the 'Online Processing' option, they will be prompted to create an account to manage the storage of their FASTQ files on the Xia Lab server. Upon login, users are prompted to upload FASTQ files via FileBrowser, and can view previously uploaded files. Next, users can initiate a processing job using either a reference (Kallisto and reference transcriptome) or reference-free approach (Seq2Fun and EcoOmicDB). After initiating a project, users are brought to the "Data Inspection & Annotation" page to select samples for quantification, label samples with experimental factors, and, in the case of paired end reads, group forwards and backwards reads from the same sample. The "Reads Mapping & Quantification" page allows users to select a reference transcriptome or use our ortholog database for read mapping together with a few quantification parameters. After job submission, the "Job Status View" page will be displayed, showing a summary of the number of completed samples and a log of the Kallisto or Seq2Fun output. At this stage, users can close the browser tab, and the job will continue to run on the server. Total processing time will vary depending on current server load, dataset size, and sample sequencing depth, but in our experience a dataset with 15 FASTQ files that are ~1GB each takes around six hours to complete. Users will receive email notices once their jobs are complete. The "Project Results" page includes a summary table of quantification statistics, PCA plots, rarefaction curves, and plots of reads quality before and after filtering. From here, users can go to the "Download Results" page to obtain the count table, feature annotation details, and other analysis information. The count table is ready for downstream analysis using the statistical modules in ExpressAnalyst.

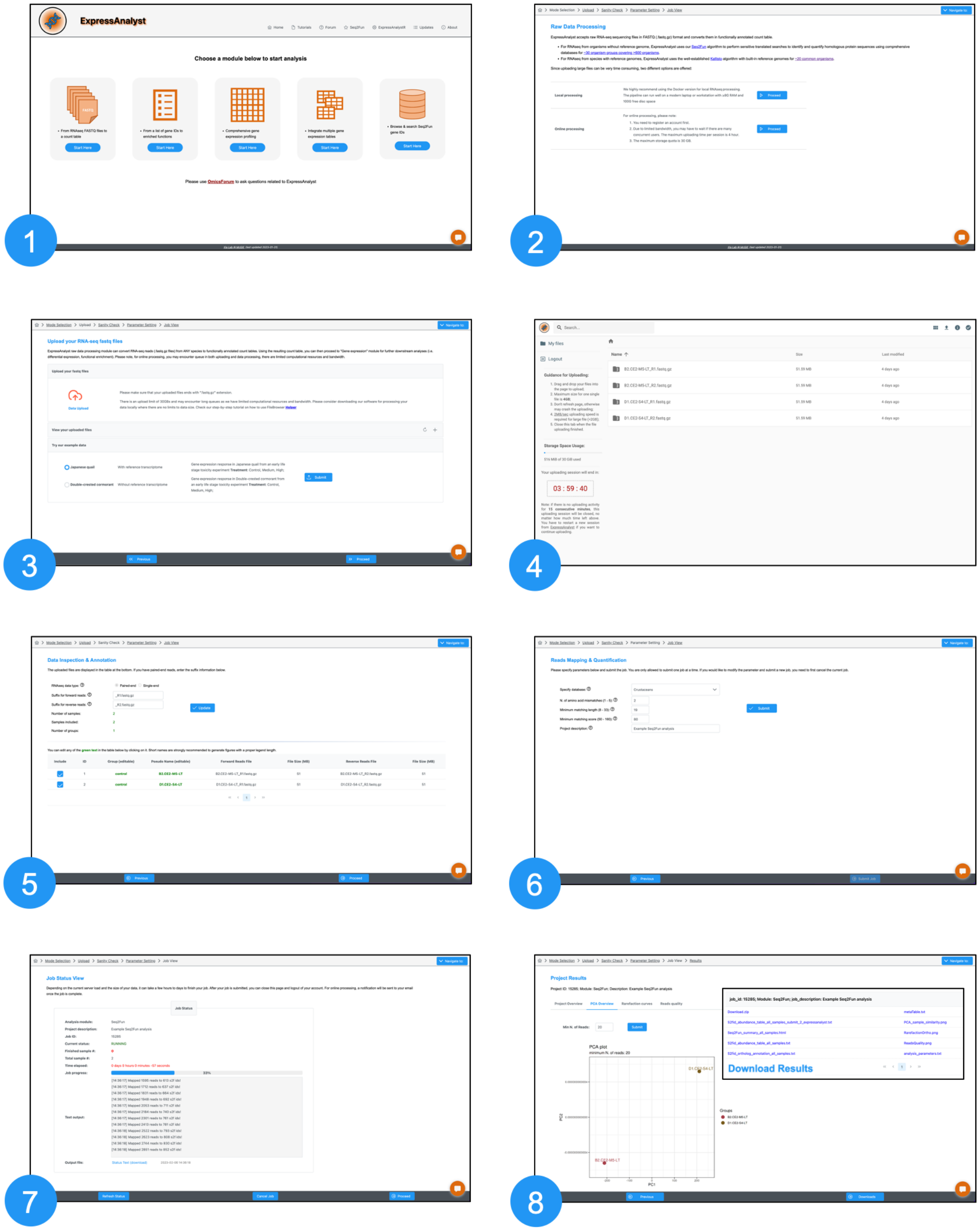

Supplementary Figure 1 - Overview of the ExpressAnalyst raw data processing interface: The raw data processing workflow includes initiation from the Module View (1), selection of online or local processing (2), file view (3), FileBrowser uploader (4), sample annotation (5), parameter setting (6), job view (7), and results via PCA, rarefaction curves, reads quality filters, and summary table (8).

Supplementary Note 2

Case Study Overview

Several datasets were used to develop, build, test, and update ExpressAnalyst. Here, we analyze two RNA-seq datasets from zebrafish (*Danio rerio*) larvae and American lobster (*Homarus americanus*) larvae, organisms with reference transcriptomes, with both Kallisto and Seq2Fun. The results are compared to show that Seq2Fun still recovers overall trends after changing the ortholog protein database that was evaluated in the original publication[1]. Note that American lobster does not have any publicly available pathway libraries for the current version of the reference genome, so functional analysis could not be performed for the American lobster Kallisto dataset. A custom fish ortholog database containing all fish sequences except for zebrafish sequences was created to analyze the zebrafish dataset.

<u>Results</u>

Case study #1: Zebrafish

The zebrafish RNA-seq data were collected as part of a previously published study that investigated the toxicity of perfluorooctane sulfonate (PFOS) compared to sodium p-perfluorous nonenoxybenzene sulfonate (OBS), a popular PFOS alternative[2]. Three groups of zebrafish embryos were exposed to 20 mg/L PFOS, 20 mg/L OBS, and 30 mg/L OBS, in addition to a fourth unexposed control group, starting at six hours post fertilization. RNA-seq profiles were measured in whole embryos at four days post fertilization. Raw FASTQ files were downloaded from NCBI's Gene Expression Omnibus (GEO) at accession GSE164074. The original study found that while immune-related genes and pathways were impacted by both OBS and PFOS exposure, a higher number were dysregulated to a higher degree by PFOS. This supports their

overall conclusion that while PFOS and OBS have a similar mechanism, PFOS is more toxic, likely because it is more bioaccumulative[3].

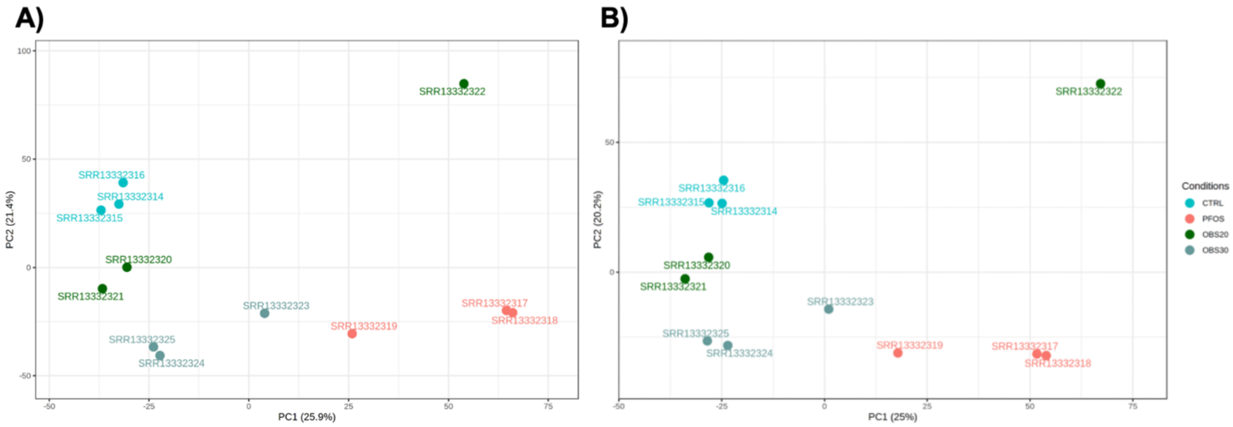

Supplementary Figure 2 - PCA of normalized zebrafish count tables: data were processed with a) Kallisto and b) Seq2Fun. These plots were generated by ExpressAnalyst.

Visual inspection of PCA plots of the normalized counts (**Supplementary Figure 2**) shows that both Seq2Fun and Kallisto captured the same variability structure among the samples, even though Kallisto mapped a higher percentage of reads (78% vs. 53%) to a higher number of features (52 518 vs. 17 186) (**Supplementary Table 1**). The higher number of reads is expected since Kallisto is quantifying multiple transcript isoforms for each coding transcript, as well as non-coding transcripts, while Seq2Fun is quantifying orthologs that do not distinguish isoforms and only include protein-coding sequences. The number of DEGs for each exposed vs. control group has the same pattern between Kallisto and Seq2Fun (PFOS > OBS30 > OBS20), with a slightly higher number in each contrast for Kallisto (**Supplementary Table 1**).

Supplementary Table 1 - Results from zebrafish DEA and GSA: Tables of the full differential expression and pathway results are in the SI_cs1_zebrasfish.xlsx supplementary file.

	Kallisto			Seq2Fun		
# Features quantified	41 345			17 186		
Reads mapping	77.49%			53.12%		
	**OBS20**	**OBS30**	**PFOS**	**OBS20**	**OBS30**	**PFOS**
DEGs	106	430	669	59	259	450
KEGG	2	1	1	1	4	10

Directly comparing the DEGs across Kallisto and Seq2Fun is challenging because of a lack of mapping between zebrafish transcripts and ortholog groups, however a cursory examination of the results shows that the top DEGs are very similar across the two software. For example, in the PFOS vs. CTRL results, the symbols “mmp9” (Entrez = 406397; Seq2Fun = s2f_0000105026), “ahsg2” (Entrez = 567406; Seq2Fun = s2f_0008962001), and “pth1a/PTH” (Entrez = 405886; Seq2Fun = s2f_0011129001) are among the top DEGs for each software, and the quantified log2FC values are nearly the same (mmp9 = 3.81, 3.78; ahsg1 = -4.64, -6.01; pth1a = 5.44, 4.57 for Kallisto and Seq2Fun respectively). The number of significant pathways is more variable between the Kallisto and Seq2Fun results, however the biological themes of the top enriched pathways are quite similar for given contrasts. For example, for both software the PFOS vs. CTRL pathways are largely related to cellular signaling and the OBS30 vs. CTRL pathways are mostly related to lipid metabolism, lipid transport, and steroid biosynthesis (**see SI_cs1_zebrafish.xlsx).**

Differences in the specific pathways flagged as enriched across the two software are expected because the Seq2Fun ortholog groups are more densely annotated than most transcriptomes.

Overrepresentation analysis is very dependent on the total number of measured genes and on the number of genes in individual pathways. Thus, even if the lists of DEGs are very similar across two analyses, the specific pathways that get flagged as enriched can vary if there are substantial differences in annotation densities, as in the case for the Kallisto and Seq2Fun analyses. This explains many of the differences in the specific pathways that were flagged as enriched between the two analysis pipelines.

Case study #2: Lobster

The American lobster RNA-seq profiles were collected as part of a study that investigated the biological impacts of exposure to heavy crude oil. One group was exposed to a water accommodated fraction (WAF) of oil at 72% concentration (WAF_72; n = 7), another to 0.39 mg/L of the polycyclic aromatic hydrocarbon (PAH) 1-methynaphthalene (positive control; n = 6), and a third group was unexposed (control; n = 6). These are new, unpublished data collected by the authors of this paper.

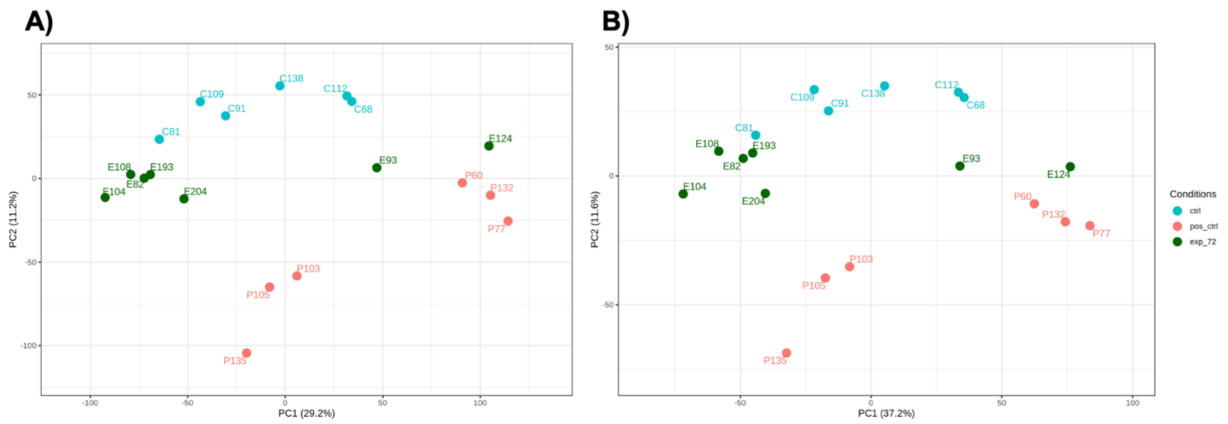

Supplementary Figure 3 – PCA of normalized lobster count tables: data were processed with a) Kallisto and b) Seq2Fun. These plots were generated by ExpressAnalyst.

The patterns with the lobster data are very similar to those with the zebrafish data. PCA plots of the normalized counts data show the same variance structure for both quantification methods (**Supplementary Figure 3**), even though more features were quantified by Kallisto and Kallisto had a higher reads and gene mapping rate (**Supplementary Table 2**). The pattern of DEGs for each contrast is also similar between Kallisto and Seq2Fun, with the positive control group having many more DEGs than the 72% WAF group. While the number of DEGs for each group is higher for Kallisto, the ratio between the two contrasts is very similar across both quantification methods.

Supplementary Table 2 - Results from lobster DEA and GSA: Tables of the full differential expression and pathway results are available in Supplementary Data 2.

	**Kallisto**		**Seq2Fun**	
# Features quantified	36 925		10 332	
Reads mapping	71.71%		29.13%	
	Pos. ctrl	*WAF 72%*	*Pos. ctrl*	*WAF 72%*
DEGs	908	50	286	13
KEGG	NA	NA	7	0

Overall, these case studies show that Kallisto and Seq2Fun give very similar results in terms of relationships between samples, as shown by PCA plots and the relative number of DEGs for different experimental groups. While the number of pathways highlighted by overrepresentation analysis is less consistent, the functional interpretation is quite similar. The lobster analysis shows one advantage of Seq2Fun for recently published genomes - no publicly available gene set

libraries exist for the American lobster transcriptome; however, we are still able to easily perform functional analysis of the Seq2Fun results (**Supplementary Table 2**).

Supplementary Note 3

Investigation of orthologs mapping to different species

It is possible that in studies containing multiple species, DEG lists could be inflated by reads from the same functional gene being mapped to different ortholog IDs for different species and being identified as differentially expressed in each case. Further analysis was performed to try to quantify the number of DEGs in the salamander case study that could be explained by this problem. The ortholog ID system is partly hierarchical – during definition, we first identified large and general orthologs, and then split these into more refined groups using an adaptive k-means clustering approach (see methods section for more details). We leveraged this hierarchical organization to assess how often the same general ortholog group A) contained specific ortholog IDs that were lowly expressed in some species and robustly expressed in others, and B) contributed multiple DEGs to the case study results.

First, we computed the average counts for each ortholog ID for each species. Next, we identified cases where the average counts were very low for one or two species (mean counts < 2) AND robustly expressed for one or two species (mean counts > 10). Next, we annotated each of these specific IDs with its more general ortholog group ID, and noted whether it was identified as differentially expressed in our case study. Finally, we counted the number of case study DEGs that were part of an a general ortholog group that had multiple DEGs with distinct species expression patterns. We found that this problem is not greatly impacting our case study, with only 83 DEGs (~3%) fitting the criteria. These results were not surprising for this dataset because there are only three amphibian species in the Seq2Fun database, none of which are

salamanders. The chances of the same functional gene being mapped to different ortholog groups is low given the taxonomic resolution of our species versus the species in the ortholog database.

Supplementary References

1 Liu, P. *et al.* Ultrafast functional profiling of RNA-seq data for nonmodel organisms. *Genome research* **31**, 713-720 (2021).

2 Huang, J. *et al.* Crosstalk between histological alterations, oxidative stress and immune aberrations of the emerging PFOS alternative OBS in developing zebrafish. *Science of The Total Environment* **774**, 145443 (2021).

3 Tu, W. *et al.* Bioconcentration and Metabolic Effects of Emerging PFOS Alternatives in Developing Zebrafish. *Environmental Science & Technology* **53**, 13427-13439 (2019).
