# Supplementary material for: ExpressAnalyst: A unified platform for RNA-sequencing analysis in non-model species
Source: Nat Commun. 2023 May 24;14:2995. doi: 10.1038/s41467-023-38785-y (PMC10209063; doi:10.1038/s41467-023-38785-y)
Supplement: Supplementary file 3 — Description of Additional Supplementary Files [file 41467_2023_38785_MOESM3_ESM.pdf]

Description of Additional Supplementary Files

File name: Supplementary Data 1
Description: An Excel workbook with differential expression analysis and KEGG gene set analysis (GSA) results for the zebrafish case study. Sheets 1-6 contain DEA results for the OBS20-CTRL (1 and 2), OBS30-CTRL (3 and 4), and PFSO-CTRL (5 and 6) experimental contrasts from Kallisto and Seq2Fun count tables respectively. Sheets 7-12 contain GSA results, organized in the same way as the DEA results.

File name: Supplementary Data 2
Description: An Excel workbook with DEA and KEGG GSA results for the lobster case study. Sheets 1-4 contain DEA results for the POS-CTRL (1 and 2) and EXP72-CTRL (3 and 4) experimental contrasts from Kallisto and Seq2Fun counts tables respectively. Sheets 5 and 6 contain GSA results for the Seq2Fun counts tables only due to lack of functional annotation of the official transcriptome.

File name: Supplementary Data 3
Description: An Excel workbook with DEA (sheet 1) and GSA results (sheets 2-9) for the salamander case study (all Seq2Fun counts). The GSA results include the GO BP (2 and 3), GO CC (4 and 5), GO MF (6 and 7), and KEGG (8 and 9) libraries for up and down regulated genes respectively.
