# Supplementary material for: ExpressAnalyst: A unified platform for RNA-sequencing analysis in non-model species
Source: Nat Commun. 2023 May 24;14:2995. doi: 10.1038/s41467-023-38785-y (PMC10209063; doi:10.1038/s41467-023-38785-y)
Supplement: Supplementary file 7 — Reporting Summary [file 41467_2023_38785_MOESM7_ESM.pdf]

nature portfolio

Corresponding author(s): Jianguo Xia

Last updated by author(s): April 5, 2023

Reporting Summary

Nature Portfolio wishes to improve the reproducibility of the work that we publish. This form provides structure for consistency and transparency in reporting. For further information on Nature Portfolio policies, see our Editorial Policies and the Editorial Policy Checklist.

Statistics

For all statistical analyses, confirm that the following items are present in the figure legend, table legend, main text, or Methods section.

n/a	Confirmed	
☐	☒	The exact sample size (*n*) for each experimental group/condition, given as a discrete number and unit of measurement
☐	☒	A statement on whether measurements were taken from distinct samples or whether the same sample was measured repeatedly
☐	☒	The statistical test(s) used AND whether they are one- or two-sided<br>*Only common tests should be described solely by name; describe more complex techniques in the Methods section.*
☐	☒	A description of all covariates tested
☐	☒	A description of any assumptions or corrections, such as tests of normality and adjustment for multiple comparisons
☐	☒	A full description of the statistical parameters including central tendency (e.g. means) or other basic estimates (e.g. regression coefficient) AND variation (e.g. standard deviation) or associated estimates of uncertainty (e.g. confidence intervals)
☐	☒	For null hypothesis testing, the test statistic (e.g. *F*, *t*, *r*) with confidence intervals, effect sizes, degrees of freedom and *P* value noted<br>*Give P values as exact values whenever suitable.*
☒	☐	For Bayesian analysis, information on the choice of priors and Markov chain Monte Carlo settings
☒	☐	For hierarchical and complex designs, identification of the appropriate level for tests and full reporting of outcomes
☒	☐	Estimates of effect sizes (e.g. Cohen's *d*, Pearson's *r*), indicating how they were calculated

Our web collection on statistics for biologists contains articles on many of the points above.

Software and code

Policy information about availability of computer code

Data collection	No software was used for data collection
Data analysis	Seq2Fun 2.02 (https://github.com/xia-lab/Seq2Fun), ExpressAnalystR 1.0.0 (https://github.com/xia-lab/ExpressAnalystR), KEGGREST 1.34.0, OrthoFinder 2.5.4, R 4.1.3, fastp 0.21.1, Kallisto 0.46.1, SRA toolkit 2.11.3, Filebrowser 1.3.6, Slurm 20.11.2, MAFFT 7.471, FastTree (no versioned releases - https://github.com/PavelTorgashov/FastTree), limma R package 3.52.4

For manuscripts utilizing custom algorithms or software that are central to the research but not yet described in published literature, software must be made available to editors and reviewers. We strongly encourage code deposition in a community repository (e.g. GitHub). See the Nature Portfolio guidelines for submitting code & software for further information.

Data

Policy information about availability of data

All manuscripts must include a data availability statement. This statement should provide the following information, where applicable:

- Accession codes, unique identifiers, or web links for publicly available datasets
- A description of any restrictions on data availability
- For clinical datasets or third party data, please ensure that the statement adheres to our policy

The salamander RNA-sequencing dataset re-analyzed in this study were deposited by the original authors in the NCBI Sequence Read Archive under accession code SRP152819 [https://trace.ncbi.nlm.nih.gov/Traces/?view=study&acc=SRP152819]. The lobster RNA-sequencing data generated in this study have been deposited in

the NCBI Gene Expression Omnibus database under accession code GSE225876 [https://www.ncbi.nlm.nih.gov/geo/query/acc.cgi?acc=GSE225876]. The zebrafish RNA-sequencing dataset re-analyzed in this study were deposited by the original authors in the NCBI Sequence Read Archive under accession code SRP299836 [https://trace.ncbi.nlm.nih.gov/Traces/?view=study&acc=SRP299836].

Human research participants

Policy information about studies involving human research participants and Sex and Gender in Research.

Reporting on sex and gender	N/A
Population characteristics	N/A
Recruitment	N/A
Ethics oversight	N/A

Note that full information on the approval of the study protocol must also be provided in the manuscript.

Field-specific reporting

Please select the one below that is the best fit for your research. If you are not sure, read the appropriate sections before making your selection.

☒ Life sciences ☐ Behavioural & social sciences ☐ Ecological, evolutionary & environmental sciences

For a reference copy of the document with all sections, see nature.com/documents/nr-reporting-summary-flat.pdf

Life sciences study design

All studies must disclose on these points even when the disclosure is negative.

Sample size	The sample size was 6-7 Stage I lobster larvae for each of the three treatment groups. This was a proof of concept study and the first time we analyzed transcriptomic responses in larval lobster. We therefore based our sample size on minimal sample size for RNA-seq experiments of 6 biological replicates, that has been suggested in the literature.
Data exclusions	No data were excluded from the analyses.
Replication	This was a proof of concept experiment that was used here to test our bioinformatics tools, not to draw biological insights. For each experimental condition we used 6-7 replicates for a total of 20 individuals, but the entire experiment was not replicated. Replication of the entire experiment was not needed in this case since we are not attempting to draw biological insights but to evaluate how the bioinformatics tools performed.
Randomization	Allocation into experimental groups was random.
Blinding	The investigators were blinded to treatment group allocation during the exposure and sample collection. The data were analyzed by a bioinformatician with no knowledge of the experimental design, beyond group labels that were needed to perform the statistical analysis.

Reporting for specific materials, systems and methods

We require information from authors about some types of materials, experimental systems and methods used in many studies. Here, indicate whether each material, system or method listed is relevant to your study. If you are not sure if a list item applies to your research, read the appropriate section before selecting a response.

Materials & experimental systems

n/a	Involved in the study	
☒	☐	Antibodies
☒	☐	Eukaryotic cell lines
☒	☐	Palaeontology and archaeology
☒	☐	Animals and other organisms
☒	☐	Clinical data
☒	☐	Dual use research of concern

Methods

n/a	Involved in the study	
☒	☐	ChIP-seq
☒	☐	Flow cytometry
☒	☐	MRI-based neuroimaging
